# Brain computer interface to distinguish between self and other related errors in human agent collaboration

**DOI:** 10.1038/s41598-022-24899-8

**Published:** 2022-12-01

**Authors:** Viktorija Dimova-Edeleva, Stefan K. Ehrlich, Gordon Cheng

**Affiliations:** 1 Munich Institute of Robotics and Machine Intelligence (MIRMI), Technical University of Munich, Munich, Germany; 2 TUM School of Computation, Information and Technology, Department of Computer Engineering, Institute of Cognitive Systems, Technical University of Munich, Munich, Germany

**Keywords:** Biomedical engineering, Brain-machine interface, Cooperation

## Abstract

When a human and machine collaborate on a shared task, ambiguous events might occur that could be perceived as an error by the human partner. In such events, spontaneous error-related potentials (ErrPs) are evoked in the human brain. Knowing whom the human perceived as responsible for the error would help a machine in co-adaptation and shared control paradigms to better adapt to human preferences. Therefore, we ask whether self- and agent-related errors evoke different ErrPs. Eleven subjects participated in an electroencephalography human-agent collaboration experiment with a collaborative trajectory-following task on two collaboration levels, where movement errors occurred as trajectory deviations. Independently of the collaboration level, we observed a higher amplitude of the responses on the midline central Cz electrode for self-related errors compared to observed errors made by the agent. On average, Support Vector Machines classified self- and agent-related errors with 72.64% accuracy using subject-specific features. These results demonstrate that ErrPs can tell if a person relates an error to themselves or an external autonomous agent during collaboration. Thus, the collaborative machine will receive more informed feedback for the error attribution that allows appropriate error identification, a possibility for correction, and avoidance in future actions.

## Introduction

Humans and machines can collaborate on a shared task to complement each other’s strengths. For example, in industrial settings, a robot may be responsible for the heavy lifting and a human may be responsible for the fine work. The differences between these actors impose challenges in the collaboration due to the dynamics of their interaction that can even lead to the human’s physical and cognitive fatigue^1^. For example, simultaneous manipulation of an object by a human and robot can lead to muscle fatigue due to suboptimal position for the human that may be caused by inappropriate action by one of the collaborators. In such situations, the human might intend to change their own position or prefer that the robot adapts. Therefore, it is important that the machine understands the human preferences. Current methods to address this problem in Human-Machine Collaboration include multimodal robot control^2^, creating models of the human^3^, and using electromyography (EMG) signals from the human as an integral signal for the robotic controller^1^. However, these methods do not answer the question if an inappropriate action was made by a human or by a machine. To foster collaboration and clarify uncertainties^2^ by understanding the human’s perception of the inappropriate action, the machine can receive electroencephalography (EEG)-based feedback from the human and learn the preferences of the human in a supervised and reinforcement learning fashion.

Brain-Computer Interface (BCI) is a system that analyzes signals from the central nervous system and translates them into machine commands. A category of BCI receives spontaneous brain signals that are not consciously controlled or modulated by the user^4^. When a person perceives an event as an error, ErrPs are spontaneously evoked in the brain, without the person’s conscious control and/or modulation, and without any prior training^5,6^. The ErrPs are characterized by distinctive positive and negative deflections (called components) in the recorded signals on the frontocentral EEG electrodes. Such passive BCI have already been suggested in the literature^7,8^ and used to give neural feedback to computer interfaces^9,10^ and robots^11–15^ for ErrPs-based adaptation and co-adaptation.

Nonetheless, it is known that different error sources elicit different types of ErrPs: (1) response to the error that the human themselves made—*self-made error*^16^, (2) response to feedback to an unrecognized self-made error^17^, (3) observation of error made by another party (a person or a machine) without any influence by the subject—*agent error*^16,18^, (4) observation of a misinterpreted action made by a machine following a user-given command, here referred as *interface error*^19^ as well as a *self-related error*, (5) *target error* that is a result of a target perturbation in an ongoing movement^20^, and (6) *outcome error* that is a result of task failure^21^.

Furthermore, the literature reports that the ErrPs characteristics differ based on the error source and the experimental design of the Human–Machine Interaction (HMI). ErrPs related to self-made vs. interface errors were studied by Spüler and Niethammer, 2015^22^ in a collision avoidance task where the subjects controlled the cursor movement on the screen. The authors could discriminate the two different sources of errors. Padrao et al., 2016^23^ studied the responses of errors visualized by a virtual avatar in two fast reaction experiments; in the first, the avatar embodied the subjects and simulated self-made and interface errors; in the second, the avatar acted as autonomous agent. They found different response patterns for the three different ErrPs. Iturrate et al., 2013^24^ found task-dependent signal variations when an object was moved towards a goal (agent errors); the goal lied either on one of the vertices of a fixed triangle around the start position, or was positioned on a horizontal/vertical grid. Furthermore, Wirth et al., 2019^25^ could distinguish similar agent errors made by a simulated robot movements by comparing the ErrPs responses; the robot (1) moved away from a target or (2) moved towards and proceeded further beyond the target. Tackling similar error events, Ehrlich and Cheng, 2019^26^ studied interface errors made by a cursor on a screen and a physical humanoid robot as a response to a human command to move/look towards one of three stimuli displayed on a screen where the error source was shown to be a factor influencing ErrPs. In the experimental design of Yazmir and Reiner, 2021^27^ where a tennis racket in a virtual tennis court was controlled by a human operating a robotic arm, error events happened when the tennis racket moved, either simultaneously and in a same direction with an applied force on the end effector of the robotic arm (congruent) or without corresponding shift of the robotic arm (incongruent). Such errors induced significantly different ErrPs. Although cursor movement error events at predicted and unpredicted states have evoked ErrPs with different latencies, Iwane et al., 2021^28^ have successfully built ErrP decoders that can transfer learning between conditions with a slight loss of performance. However, not all unexpected events evoke ErrPs. Si-Mohammed et al.^29^ found indication that events that do not affect successful task completion may not evoke ErrPs.

The different characteristics of ErrPs can be used as a more informed feedback to any machine collaborating with a human that detects human intention, and has autonomy in solving the shared tasks. When a human and a machine work as partners, an event might cause ambiguity in the responsibility perceived by the human partner^30,31^. Understanding the responsibility as perceived by the human is important to inform the machine for the future steps. Previous literature has shown that the sense of responsibility influenced the neural mechanisms of outcome processing that led to higher amplitude in the measured response for higher responsibility^32–35^. To accommodate the human preferences, ensuring satisfactory sense of agency in the human partner^36^, an ErrP evoked by perceived agent error would inform the machine to update the decision policy. On the other side, self-related ErrP would signal that no update is required. In shared control settings, where the system identifies the user intention and helps to execute the task faster^37^, self-related ErrP would inform the system about the misinterpretation. In contrast, agent-related ErrP would inform the system about the correctly identified user intention and that the error is in the assistance.

**Figure 1:**
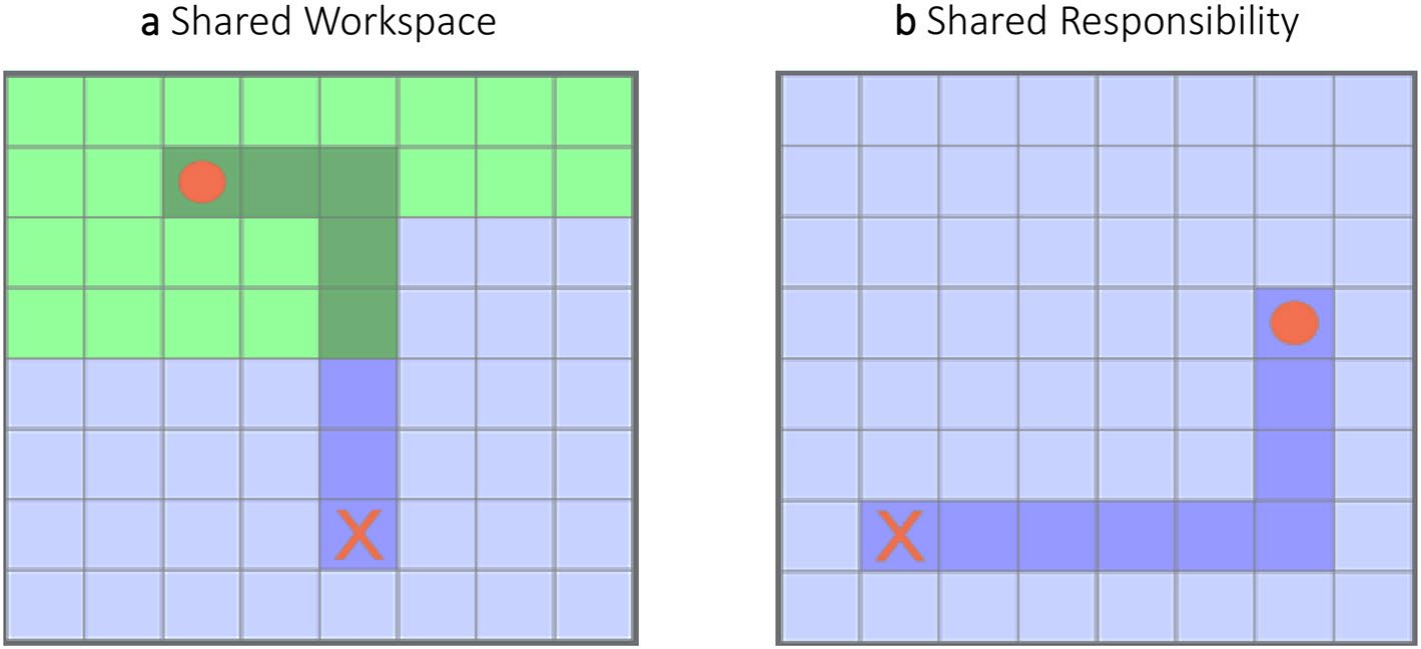
Collaboration scenarios: (**a**) Shared workspace scenario where the workspace is divided into two areas, and each actor can control the object movements only over one area—either colored in green or blue. This scenario simulates collaboration on the same task with object hand-over between a human and an agent; (**b**) Shared responsibility scenario where both actors control the movement of the object in only two directions (e.g. only up and left) out of the four possible directions (up, down, left, and right). This scenario simulates collaboration on the same task, where both the human and the agent physically and sequentially interact with the object at the same time. The background was randomly switching between green and blue.

With this work, we address the questions if responses to *self-* and *agent-related* errors yield different characteristics, and if such potential differences can be distinguished. For that, we designed a Human-Agent Collaboration (HAC) experiment that simulates two levels of collaboration and contains errors both when the human is in control and when the human is observing. Such errors are expected to evoke ErrPs^7,12,13^. We contribute to the line of BCI research on ErrPs evoked in settings where humans interact with machines, or agents in general, by showing that self- and agent-related errors are separable.

## Results

### Experiment overview

The HAC experiment (shown in Fig. 1) was in the form of a grid-world game where the human subject and the programmed autonomous agent worked in a shared workspace with a shared task^38^ simulating assembly task where the order of the steps is preassigned. The task was to collaboratively move an object from a start position by moving it up, down, left, or right one tile at a time along a marked trajectory to a marked goal by stepped on each marked tile in the assigned order. The trajectory served as a clear indicator for the next correct step. Throughout the experiment, it was at all times clear to the subjects that either only they or only the agent could control the movement of the object. The actors achieved the shared task across two collaboration levels: (1) Shared Workspace Scenario (SW) (shown in Fig. 1a) that contained complementary grid areas, in blue and green colors, where the human had independent full control only over one of them and the agent only over the other. This scenario modelled a collaboration on a shared task where one of the actors hands over the object to the other actor, and (2) Shared Responsibility Scenario (SR) (shown in Fig. 1b) with complementary control across the grid where each of the actors could control the object’s movement in only two out of the four possible directions. This scenario modelled a collaboration on a shared task where both actors simultaneously physically interact with the object. To avoid color-sensitive ErrPs responses^39^, the blue and green colors of the grid were changed randomly. We collected EEG data from 11 subjects and the following results are from 10 of them. The results from the omitted subject are presented in the Appendix.

The terminology that we use for the experiment is the following: a *trial* is one discrete movement from one tile to an immediately neighbouring tile; an *episode* is the set of trials required to bring the object from the start position, along the trajectory, to the goal; a block is a set of 13 episodes of the same scenario, each with different start position and goal.

#### Error definition and occurrence

Errors were introduced by deviation from the trajectory, or the given control. On the one hand, an *interface error* occurred when the object moved towards a direction different from the one given as a key press (up, down, left, or right arrow key) by the subject. The meaning of the word *interface*, that explains the error, is solely in the context of a computer receiving an instruction from the user, excluding the context of a computer giving information to the user. On the other hand, an *agent error* occurred when the autonomous agent was in control (and the subject was just observing), and the object did not move towards the next tile of the marked trajectory. The wrong direction was uniformly and randomly selected from all three possible wrong directions. The error probability ranged between 25% and 35%. However, no two sequential errors could occur. Therefore, the average error rate was 23.3% ± 0.9% from a total of 2322.4±85.4 trials.

### Electrophysiological characteristics

**Figure 2:**
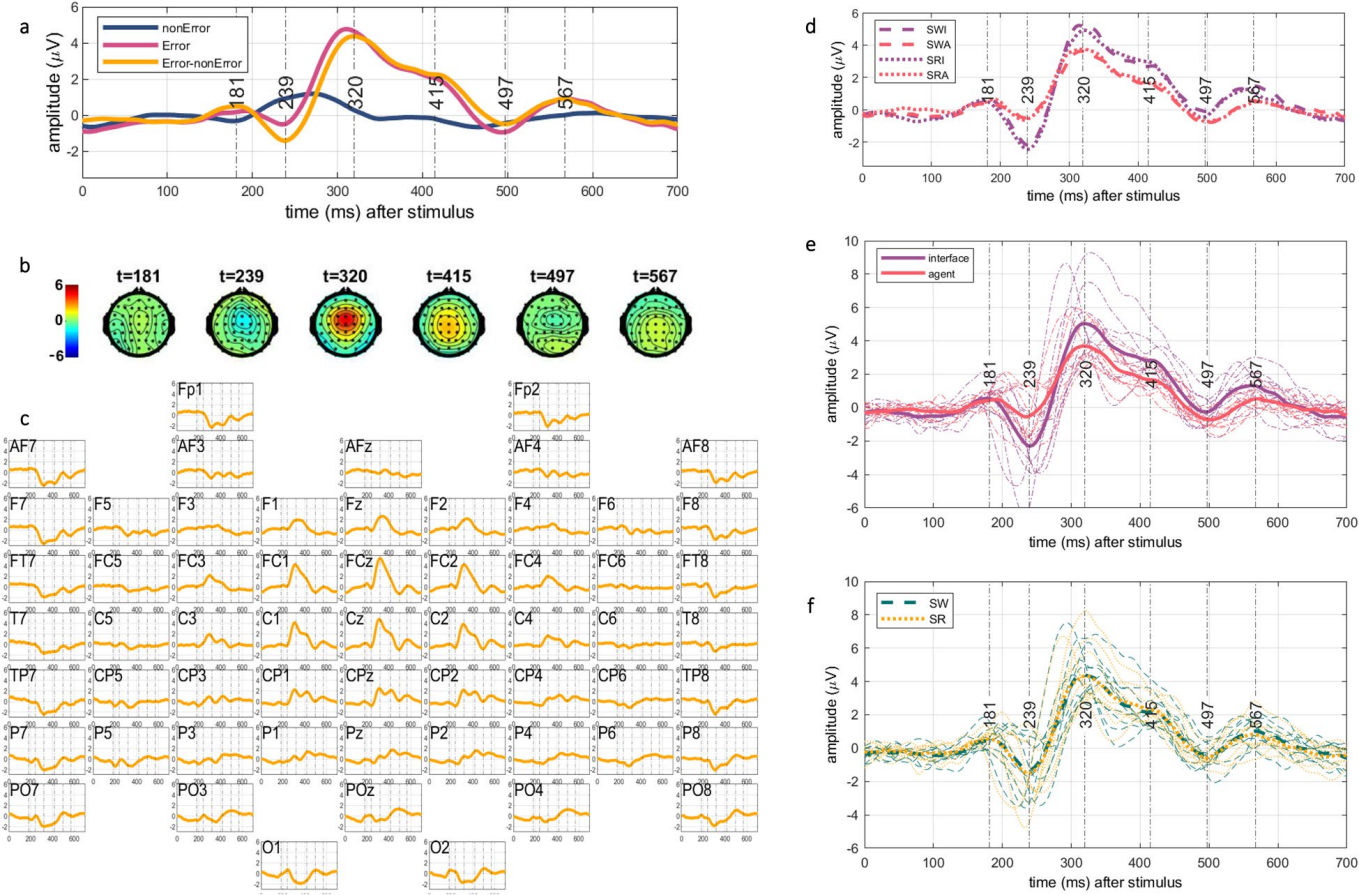
Grand average from all subjects and all trials: (**a**) the grand averaged signals on the Cz electrode for all error (pink) and non-error (dark blue) trials and their difference (orange) across all conditions in a time window between 0 and 700 ms after the object movement. Positive and negative peaks of the components can be observed around 181 ms, 239 ms, 320 ms, 415 ms, 497 ms, and 567 ms; (**b**) the topographic maps of the difference between the grand average of the error minus non-error trials for the positive and negative peaks in (**a**); (**c**) the difference of the grand averaged error and non-error signals across all electrodes; (**d**) differences in the amplitude of the responses for the different sources (interface in purple and agent in red) of the errors in the different scenarios. The dashed signals represent the grand averages of the Shared Workspace (SW) scenario, whereas the dotted signals represent the grand averages of the Shared Responsibility (SR) scenario; (**e**) the response for the interface errors (thick purple) at the peaks has higher amplitudes compared to the response for the agent errors (thick red). The thin dash-dotted lines show the averaged trials per subject; (**f**) the overlapping responses to the different scenarios (SW in green dashed lines, SR in orange dotted lines). The thick lines show the grand-averaged trials, and the thin lines the averages per subject.

Because of the fronto-central distribution of Error-related potential (ErrP), we analyzed the temporal characteristics of the components, their polarity, and peaks on the Cz electrode. Figure 2a shows the Cz electrode temporal dynamics of the grand-averaged trials where the movement of the object was correct, the movement of the object was erroneous, and the difference between the two. Figure 2b shows the topographical maps for the difference between the grand-average of the error minus the grand-average of the non-error trials. Observing the Cz channel time course and the topographical maps we can recognize that there is a small frontocentrally-located positive peak around 180 ms (in the further text referred as the P200 component), negative frontocentral deflection between 200 ms and 250 ms (referred as the N200 component), positive part between 250 ms and 450 ms with a peak around 300 ms (referred as the P300 component), centrally-located negative deflection between 450 ms and 500 ms (referred as the N450), and a small centroparietal positivity between 500 ms and 600 ms (referred as the P600 component). The P300 component seems to constitute of P3a and P3b components, the peak around 300 ms (P3a) is present more frontally and the peak around 400 ms (P3b) more parietally. Figure 2c shows the grand-average temporal dynamics recorded on all electrodes.

The temporal dynamics across the averaged ErrPs of both scenarios (SW and SR) and both error sources (error performed by the interface or by the agent) across all subjects are displayed in Fig. 2d-f that depict the differences in the components in time domain. The components exhibit visual differences in the amplitudes for the different sources.

### Network dynamics

Past fMRI and EEG studies have continuously shown that the Anterior Cingulate Cortex (ACC) is involved in errors recognition^8,40,41^. For the sake of simplicity, we specifically focused on ACC analysis since its activation was previously reported in context of interface and agent errors^7,24^.

#### Relationship with the experimental factors

We analyzed if the experimental factors modulated the activity of the ACC. Specifically, if the estimated Average Absolute ACC Activation (AAACC) prior and posterior to the Cz-electrode peaks of each component originate from the same distribution for the different error sources and scenarios across subjects. We computed the AAACC in a − 25 to 25 ms window around the peaks of the P200, N200, N450, and P600. Instead of computing two separate averaged values for P3a and P3b, we used a fixed window between 280–450 ms capturing the onset and offset time of the P300 component. A three-way Analysis of Variance (ANOVA) test was performed on the AAACC of the five components (P200, N200, P300, N450, and P600). The test factors were the scenarios, sources of errors, subjects, and interaction between them. Bonferroni correction was applied to correct the common p-value of 0.05 by dividing it by 5 (since five tests were made). Hence, the hypothesis was rejected for p-value below 0.01. We computed the Spearman correlation coefficient between AAACC activity of the components, and scores from the questionnaires for each subject individually, and difference between the scores for the perceived intelligence and perceived safety were tested for correlation with the average amplitude surrounding the peaks of the components. Table 1 shows the relationship between the source types (interface or agent), scenarios (SW or SR), the subjects, and the AAACC around the peaks of each component. It reveals that there are significant differences between the components for different sources (interface or agent) and for each subject individually. Furthermore, the interaction between the source and the subject is significant for the P200 and the P600 component.

**Table 1:** Results of the ANOVA test on the effect of the factors of the experiment (source: interface or agent, scenario: SW or SR, and the subjects) and their interaction on the average absolute Anterior Cingulate Cortex (ACC) activation around the peaks of the ErrP components.

Factors	P200	N200	P300	N450	P600
Source	**2.81e−03**	**3.27e−03**	**4.92e−03**	**2.22e−03**	**9.96e−04**
Scenario	4.84e−01	4.61e−01	3.78e−01	4.80e−01	4.62e−01
Subject	**3.65e−06**	**5.68e−06**	**1.48e−05**	**6.26e−06**	**1.58e−06**
Source × scenario	1.90e−01	3.08e−01	2.71e−01	1.00e−01	1.29e−01
Source × subject	**9.66e−03**	1.02e−02	2.44e−02	2.93e−02	**8.13e−03**
Scenario × subject	3.75e−01	2.72e−01	2.71e−01	3.00e−01	6.17e−01

Statistically significant values are in bold.

#### Relation with personality traits

To evaluate if the participants’ personality traits and perception of robots collaboration have effect on the ErrPs, the subjects filled in two questionnaires: (1) the Big Five questionnaire^42^ which evaluates five personality traits (extroversion, conscientiousness, neuroticism, and openness to experience), and (2) the Godspeed Questionnaire^43^ that evaluated the perceived intelligence of robots and safety that shall capture the subjects’ readiness for collaboration in regard to robots and agents. The subjects completed the Godspeed Questionnaire both before, to rate the impression of robots in general and after the experiment, to rate the impression of the agent. The comparison between the two shall indicate how the experiment affected the perception. We inspected whether there are correlates between the results of the questionnaires and the factors of our collaboration experiment. Statistically significant correlation was found with the neuroticism score from the Big Five Questionnaire, which results are shown in Table 2. For all components, correlation around 0.65 was found with the AAACC activation of the interface errors. Correlation of above 0.7 was found for the difference (interface minus agent) in the AAACC.

**Table 2:** The p-values and the Spearman correlation coefficients between the AAACC during the components and the neuroticism scores of the Big Five Questionnaire.

Components	ACC activation during interface errors		Difference between the ACC activation during interface and agent errors	
	p	Correlation coefficient	p	Correlation coefficient
P200	4.70e−02	0.64	1.66e−02	0.73
N200	4.70e−02	0.64	1.41e−02	0.74
P300	3.92e−02	0.66	1.08e−02	0.76
N450	3.45e−02	0.67	8.88e−03	0.77
P600	4.70e−02	0.64	1.66e−02	0.73

### Classification of single trials

#### Selection of features

We used the forward feature selection method with a linear Support Vector Machine (SVM) classifier to rank the electrodes that achieve the best sensitivities

$$\begin{aligned} S_{type} = (S_{error} + S_{non-error}) \cdot \frac{1}{2} = (\frac{T_{error}}{T_{error} + F_{non-error}} + \frac{T_{non-error}}{T_{non-error} + F_{error}}) \cdot \frac{1}{2} \cdot 100\% \end{aligned}$$

where $T_{error}$ is the number of correctly predicted error trials, $F_{non-error}$ the number of error trials that were incorrectly predicted as non-error trials, $T_{non-error}$ is the number of correctly predicted non-error trials, and $F_{error}$ the number of non-error trials incorrectly predicted as error trials, and

$$\begin{aligned} S_{source} = (S_{interface} + S_{agent}) \cdot \frac{1}{2} = (\frac{T_{interface}}{T_{interface} + F_{agent}} + \frac{T_{agent}}{T_{agent} + F_{interface}}) \cdot \frac{1}{2} \cdot 100\% \end{aligned}$$

where $T_{interface}$ is the number of correctly predicted interface errors, $F_{agent}$ the number of interface errors incorrectly predicted as agent errors, $T_{agent}$ the number of correctly predicted agent errors, $F_{interface}$ the number of agent errors incorrectly predicted as interface errors.

**Figure 3:**
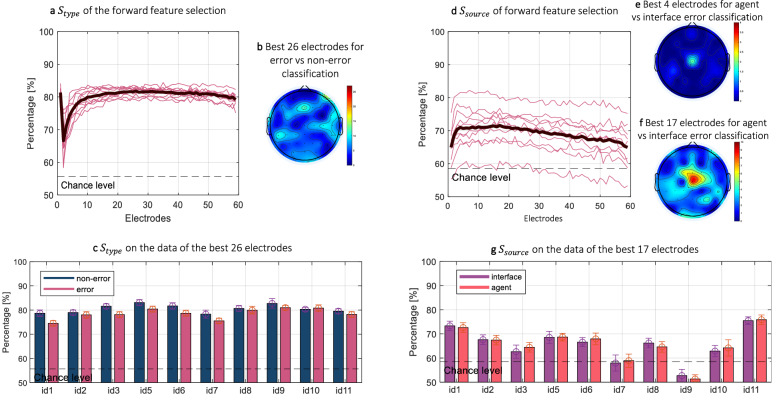
Electrode selection and the respective sensitivity: (**a**) the change of $S_{type}$ as the data from each of the additional electrodes was added to the classifier; (**b**) the distribution of the average of the best 26 electrodes; (**c**) the achieved $S_{type}$ non-error (dark blue) vs error (pink) for each subject on the data of the average best 26 electrodes; (**d**) the change of $S_{source}$ as the data from each of the additional electrodes was added to the classifier; (**e**) the distribution of the average of the best 4 electrodes that achieved the second-best average $S_{source}$; (**f**) the distribution of the best 17 electrodes that achieved the best average $S_{source}$; (**g**) the achieved $S_{source}$ for each subject on the data of the average best 17 electrodes, interface errors in purple, and agent errors in red.

The feature selection, i.e., electrode selection, started by training SVM classifiers with linear kernel for the data of each of the electrodes separately in the first iteration. The sensitivity of each class was calculated, and the electrode $E_1$ that achieved the best sensitivity was fixed for the next iterations. In the second iteration, the already selected electrode $E_1$ was used in combination with every other electrode $E_i$ to train SVM classifiers with data from two electrodes. The combination with the second-best electrode $E_2$ gave the best sensitivity. The iterations continued until all electrodes were ranked. The electrode selection was done for error trials vs non-error trials where the electrodes were ranked based on the highest sensitivity $S_{type}$, and interface errors vs agent errors where the electrodes were ranked based on the highest sensitivity $S_{source}$. Figure 3a shows the change in $S_{type}$ as the data of additional electrodes were added to the SVM classifier. We can observe that $S_{type}$ was around 80% for the data from only one electrode, and it dropped more than 10% when the data of a second electrode was added. On average across all subjects, when the classifier received the data from each one electrode, it achieved $S_{type}$ of $79.4\% \pm 0.23\%$. The best $S_{type}$ was achieved with the data from 26 EEG electrodes (AF8, Fz, FT7, CP5, C4, F8, FC6, FT8, F1, FC3, TP8, C6, C2, CP6, CP3, O2, CP1, F2, T7, PO3, F5, POz, F4, O1, P7, FC1). Figure 3b shows the distribution of the best 26 electrodes.

The best $S_{source}$ was achieved with the data from 17 EEG electrodes (Cz, CP3, FCz, AF8, CP6, FC2, AFz, P5, FT8, T7, FC1, C3, CP5, P4, C4, FT7, CP4), and the second best with the data from only 4 EEG electrodes. For easier interpretability, we show only the first 4 electrodes for each subject in Table 3. Table 3 also shows the best result for each subject and the number of required electrodes to achieve it. Furthermore, Fig. 3d shows the change in $S_{source}$ as the data of additional electrodes were added to the classifier, Fig. 3e shows the distribution of the best 4 electrodes, and Fig. 3f shows the distribution of the best 17 electrodes. We can observe that the central electrodes have contributed the most towards higher $S_{source}$.

**Table 3:** $S_{source}$ on the data of the top 4 electrodes for each subject separately.

	Subject id	1	2	3	5	6	7	8	9	10	11	Average
Top 4 electrodes	$S_{source}$ (%)	77.21	75.41	69.15	71.34	69.04	65.99	73.23	59.34	67.46	81.57	70.97
	Electrodes	Cz	Cz	CP3	T7	FCz	FT8	Cz	FC2	P5	Cz	Cz
		AF8	CP4	FT7	FC1	CP6	Cz	CP5	P4	CP3	C3	CP3
		C2	F4	TP7	AFz	C4	F3	AF8	AFz	P1	FC6	FCz
		PO4	C6	FC4	P2	T8	PO3	C4	FCz	P8	CP6	AF8
Best result	$S_{source}$ (%)	78.21	76.42	70.77	75.31	72.59	68.27	73.79	60.80	68.04	82.22	72.64
	# Electrodes	3	11	30	18	16	23	8	18	17	8	15.20

#### Prediction of errors

**Figure 4:**
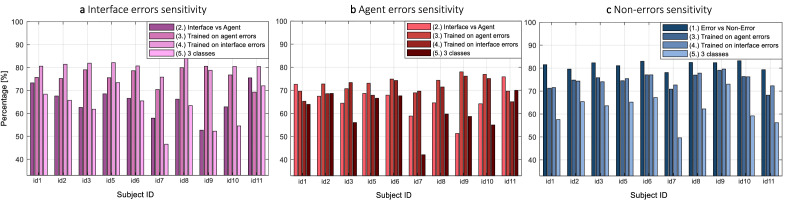
SVM classification sensitivity results per subject for each of the five different classifiers that were trained: (**a**) $S_{interface}$ in purple; (**b**) $S_{agent}$ in red; and (**c**) $S_{non-error}$ for each of the SVM classifiers. The classifiers (1)–(4) were solving a two class problem: (1), (2), and (3) were solving a non-error vs error problem. The error set for training and validation consisted of both agent and interface errors for (1). The classifier (3) was trained on agent errors and validated both for interface (shown in **a**), and agent (shown in **b**) errors, whereas (4) was trained on interface errors. The classifier (2) was trained to distinguish interface and agent errors. The classifier (5) was solving a three class problem of decoding whether the event was non-error, interface error, or agent error.

After selecting the top 17 electrodes, we trained 5 SVM classifiers: (1) discriminating error from non-error events, (2) discriminating between interface errors and agent, (3) discriminating between error and non-error trials where the error trials were made only by the agent, (4) discriminating between error and non-error trials where the error trials were made only by the interface, and (5) three classes, one for non-error trials, one for interface errors, and one for agent errors. Table 4 shows the averaged results for each of the classifiers and the classes, as well as the respective chance level. Figure 4 shows the $S_{interface}$, $S_{agent}$, and $S_{non-error}$ for each classifier and for each subject. The results show that the two-class problem of detecting the error source leads to lower sensitivity in comparison to the detection of the event type. Table 4 and Fig.4c show that the $S_{non-error}$ is not dependent on the source of errors on which the classifier is trained. However, the results suggest that the interface errors can be better classified than the agent errors. The three-class problem of the (5) classifier, although leading to expectantly lower sensitivities, has results above the change level like all other classifiers. Similarly to Figs. 3 and 4 shows that the subjects id 7 and id 9 have sensitivity close to chance level.

**Table 4:** The average of the sensitivity for each condition and each trained SVM classifier.

	(1) error vs non-error	(2) interface vs agent	(3) trained on agent errors vs non-errors	(4) trained on interface errors vs non-errors	(5) interface errors vs agent errors vs non-errors
$S_{interface}$ (%)		$65.38 \pm 2.3$	$76.12 \pm 2.76$	$80.66 \pm 2.38$	$62.36 \pm 6.58$
$S_{agent}$ (%)		$65.59 \pm 2.2$	$72.9 \pm 2.32$	$70.68 \pm 2.26$	$60.84 \pm 8.31$
$S_{error}$ (%)	$78.52 \pm 2.17$				
$S_{non-error}$ (%)	$80.56 \pm 1.69$		$74.48 \pm 3.37$	$75.1 \pm 2.6$	$61.92 \pm 6.56$
Chance level (%)	55.7	58.53	58.53	58.53	48.34
Average (%)	$79.54 \pm 1.08$	$65.49 \pm 6.8$			$61.7 \pm 6.6$

## Discussion

In an experimental setup of HAC with erroneous control, we collected EEG data from 11 subjects and analyzed the response (of ten subjects) to self- and agent-related errors within two different collaboration levels, SW where the human controlled the movements of the object in a separate area of the shared workspace and could not interfere with the autonomous agent, and SR where the human had limited control across the whole shared workspace and could take charge of a movement between two controls from the agent. The characteristics of the averaged signals on the central Cz electrode are in line with the ErrPs reported in the literature^9,19^. They have a positive peak followed by a negative peak along with a broader positive signal that has two bumps and is again followed by a negative and a positive peak, all of which being within the 150 ms to 650 ms interval after the occurrence of the event (movement of the object). These peaks exhibit different amplitudes, which are greater for the different sources (interface and agent errors).

To obtain a better understanding of the neural origins of the observed EEG effects, we investigated the temporal activation at the source level and for simplicity, focused only on the analysis of the estimated activation in the ACC, area known to be involved in recognition of errors^44^. The results showed no effect of the different collaboration levels, but showed a statistically significant effect of the source of error. The source of the error was a modulating factor since the responses come from separate distributions. These results are interesting and prompt the question for further research if independently on the circumstances under which a system misinterprets a command given by the user, the evoked response would be stronger than in case when the user observes an autonomous system performing an action that is unexpected. Regarding the collaboration levels, SW had very clear and strict separation of the responsibility in control of the movement between the human and the agent—in the area that the human was in control, the agent had no influence on the movement, and vice versa. The responsibility was switched only at the border. In comparison, the SR scenario required higher level of readiness for taking over the control from the agent since the responsibility for the future trials cannot be predicted ahead of time. The reasons are: (1) the assigned control over two out of the four possible directions of movement across the workspace that was not visually displayed during the episodes, and (2) the errors in the experiment. Despite the differences in the scenarios, the results of the ANOVA test show that the responses for these two scenarios come from the same distribution. Therefore, we can infer that the higher alertness required for the SR scenario was not factor that modulated the ACC activation in our experimental design presented in this work. Furthermore, due to the task relevance, we suggest that the agent control was emotionally significant experimental condition in both scenarios^45,46^.

Furthermore, we found statistically significant positive correlation between the neuroticism and the difference between the responses to the different sources of errors showing tendency of bigger ACC activation for interface errors relative to agent errors when the neuroticism score of the subject is higher. Positive correlation between neuroticism and the ACC was already reported in the literature^47^. In particular, Haas et al.,^47^ found higher ACC activation during trials of high emotional conflict, compared with trials of low emotional conflict. Therefore, our results suggest that people with a higher neuroticism score tend to experience higher emotional conflict for the self-related error as to the agent-related errors. These results do not immediately suggest that the estimated ACC activation is specific to the interface and agent errors. It may be that the subjects with higher neuroticism score have higher ACC activation due to emotion-induced stimulation or other spontaneous activation related to increased arousal^48^. Therefore, a larger dataset is required to consolidate this finding. We suggest that this study can be used as a basis for more psychology-focused study on brain responses in the context of HAC tasks with additional physiological measures such as electrocardiogram and electrodermal activity to better study the relation of the emotional significance of the experimental stimuli, the neuroticism score and the activation specificity in regards to the evoked ErrPs.

We could correctly predict error and non-error events in $79.54\% \pm 1.69\%$ of the trials using the data from the 26 channels that provided the best features across all subjects. This result is comparable with other ErrPs studies. On average, we could correctly predict interface and agent error events in 65.49% ± 6.8% of the cases based on the data from the 17 channels that achieved the best overall prediction. The results from subject id 7, and subject id 9 were below chance level; without them the average of the sensitivity of interface and the sensitivity of agent errors was 70.7% ± 4.34%. Please note that as displayed in Table 3, $S_{source}$ for subject id 7 was above, and for subject id 9 slightly above the chance level of 58.53% when the channels were selected based on the subjects’ individual EEG. In a three-class problem, the average sensitivity was 61.7% ± 6.6%. It is to be expected that the sensitivity can be higher with a selection and fine-tuning of another classifier. These results indicate that independently on the collaboration scenario, an erroneous event that the user relates to the self can be distinguished from one made by an agent. Such differentiation is significant for intelligent systems that identify user intention and autonomously take action by understanding the source of the error which in turn would suggest possible appropriate next actions for correction. Previous literature shows promising results that even for subjects with low ErrPs-classification accuracy, the performance of hybrid BCI could be improved^49–51^. Computationally, we have shown that even slightly above chance-level accuracy yields improvements in human-robot co-adaptation^52^.

We also tested the transfer learning between sources of errors. An SVM classifier trained on interface errors predicted agent errors with sensitivity of 70.68% ± 2.26% which was worse than predicting other unseen interface errors (80.66%±2.38%). The achieved sensitivity of predicting interface errors was slightly better than if a classifier was trained on all non-error vs all error trials (sensitivity of 77.04% ± 2.38% for the error events). However, interestingly, a classifier trained on agent errors predicted interface errors with sensitivity of 76.12% ± 2.76% which was higher than the sensitivity for predicting agent errors 72.9% ± 2.32%. These results indicate that a classifier trained only on one source of error can generalize well to be able to detect the other source of error. However, independently on the source of error that the classifier will be trained, it would be more sensitive to ErrPs with higher component amplitudes.

We suggest that future co-adaptation^13^ and shared-control BCI systems^15,53^ may study the possible benefits from the detection of different self- and agent-related ErrPs. We suggest that, for subjects that have distinct self- and agent-related ErrPs, detecting the self-related ErrPs would improve the reliability (by correcting the misinterpreted user intention) and accuracy (by updating the user intentions decoder through time), and the detection of agent ErrPs would allow the system to better satisfy the user needs. The distinction between the two is necessary for a system to identify the stage at which an error occurred—at the level where the user intent is detected or at the level where the system executes the decoded intent. To support the results that we presented, it would be beneficial to do a follow-up study with a robot as a collaborator^54^ since it was earlier found that misinterpretations of user commands displayed on a screen or used to control humanoid robot evoke ErrPs with different characteristics^26^, and that the response in collaboration with a robot is different from the response in collaboration with a human^55^.

## Methods

### Participants

Eleven right-handed participants (27 ± 3.82 old, 7 with normal and 4 with corrected-to-normal vision, 5 with previous experience with EEG experiments) participated in the study approved by the Ethics Committee of the Technical University of Munich with reference number 80/20 S-KH. All experiments were performed in accordance with the principles in the Declaration of Helsinki. All declared their informed consent in written form. They were not checked if they take any medications. The number of participants is comparable with similar studies.

### Experimental design

Figure 1 shows the game (programmed in Python 2.7 using PyGame) that simulated two collaboration levels in two respective scenarios. The scenarios consisted of episodes with a task to move an object from its start position along a marked trajectory to a marked goal; all randomly initialized on an 8x8 grid. The object represented by a circle and the goal represented by an “X” were colored red (shown on the upper parts of Fig. 1). The grid was light blue or light green, and the color of the marked trajectory was dark blue or dark green. The workspace division in SW was between 37.5% and 62.5% for each area. Each area was represented either in green or blue. The grid color and the responsibility for the movement directions in SR were randomly selected for each block. The object could move in four directions (up, down, left, and right). In an ambiguous situation in SR where the next correct trajectory tile was diagonal to the current position, the priority was in clockwise direction (up had the highest priority and left the lowest). The subjects were expected to understand this order of priority during the experiment. The Supplementary Materials contain example videos for both scenarios.

### Stimuli and apparatus

In a quiet room, the participants sat comfortably on a chair at 2 meters distance to a 24” display with 60 Hz refresh rate. The agent movements were automatic, whereas to make a step, the subjects pressed the arrow keys on the keyboard in front of them. To prevent the subjects from habituation to the stimuli^56–58^ by predicting the moment of agent-controlled object movement, and to avoid frequency- sensitive results^59–61^, the inter-stimulus interval (ISI) between two movements was randomly changing. It was selected in the range between 700 ms and 900 ms before each episode and kept the same within the episode. The program did not register any key-pressing before the ISI. Minimum 7 tiles separated the start position and the goal which were never in the same row or column (requiring at least one turn, which was designed to increase the collaboration by minimizing the number of possible episodes where the object is controlled either only by the human or only by the agent). The error probability ranged between 25% and 35%. The error rate was uniformly and randomly selected at the beginning of each block and kept the same throughout the block. We introduced the varying error probability to prevent the subjects from getting used to the errors frequency and predicting their occurrence. However, before the EEG data collection started, the subjects practiced both scenarios by completing 5 episodes from each. To make the subjects aware that errors occur in the experiment, the error probability during the practice was fixed to 20%.

### EEG data acquisition

The data from the subjects were collected using a battery-driven Brain Products actiChamp amplifier with 65 active gel-based electrodes and 1000 Hz sampling rate and were transferred via USB to a recording PC (Intel Core i5CPU 750@2.67 GHz, 64-bit Windows 7). The data recording was done using BrainVision PyCoder software, version: 1.0.9. Fifty-nine electrodes (Fp1-2, AFz, AF3-4, AF7-8, Fz, F1-8, FCz, FC1-6, FT7-8, Cz, C1-6, T7-8, CPz, CP1-6, TP7-8, Pz, P1-8, POz, PO3-4, PO7-8, O1-2) were arranged according to the 10–10 system^62^, Fpz was ground, TP9 and TP10 were reference electrodes, and three electrodes recorded the EOG activity on the forehead and the left and right outer cheekbones. All impedances were below 60 k$\Omega$ except of some (reported in Table A1 in the Appendix) that were interpolated.

### Experimental protocol

All subjects followed the same protocol. They completed the questionnaires and received all instructions during the EEG electrodes placement. Afterwards, they executed a test block of the SW scenario and SR, each having 5 episodes. The subjects were instructed to sit relaxed, avoid blinking and limbs tension, and rest their wrists on the desk to avoid movements. To keep the subjects engaged and have enough trials for statistically relevant results, the experiment was designed to have above 2000 trials and blocks below 3 mins, resulting in 12 blocks (six in each scenario) with 13 episodes each. Each episode had different start position and goal with new trajectory. The order of the scenarios was alternating to avoid any possible effect from the order on the ErrPs. The breaks between blocks were controlled by the subjects. The subjects completed the second questionnaire by the end of the last block. The duration of the experiment including the breaks was around 75 mins, together with the preparation before and after around 3.5 h. The participants received a honorarium of 8 euros per hour.

### Data analysis

#### Preprocessing

To remove low frequency drifts, power noise, and high frequency muscular activity, the data were band-pass filtered using Hamming windowed sinc FIR filter with low cutoff of 1 Hz, high cutoff of 40 Hz, and transition band of 1 Hz. To discard any noise corruption, we removed the collected data from the breaks, and the noisy channels (that contained muscle movement artifacts, often the case for the electrodes T7 and T8 after the breaks) by visual inspection. Next, EOG artifact removal was done using the method by Schoegl et al.^63^ and the data were re-referenced to a common average. The rejected channels were interpolated using spherical interpolation method. Finally, the trials that stayed noisy (having artifacts such as muscle movement, and EOG not cleaned with the EOG artifact removal) were rejected by visual inspection. The subject with id4 had many noisy trials leaving 805 non-error and 236 error trials after rejection, whereas all other subjects had 1774±48.92 non-error and 520.5 ± 27.23 error trials (Table A2 in the Appendix shows the numbers for each individual subject). Therefore, id4 (Table A3 in the Appendix) was discarded from the analysis across subjects and the individual results are reported in the Appendix. The preprocessing was done in Matlab R2019b, using EEGLAB 2019.0^64^ and self-written code.

#### Data epoching and averaging

Since the minimal ISI between two subsequent movements was 700 ms, the time before stimulus belonged to the end of the previous stimulus and the time after 700 ms belonged to the next. To avoid any overlap with the surrounding stimuli, the data were segmented into epochs between 0-700 ms after object movement, both for error and non-error movements. Grand averages of the trials were computed according to the type of the event (error or non-error), the scenario (SW or SR), and the source (agent or interface).

#### Component characteristics

Under the assumption that the signal-to-noise ratio across conditions is comparable, Aggregate Grand Average from Trials (AGAT) is a robust^65^ method of finding the Region of Interest (ROI) by computing the average on all trials from all subjects and all conditions. Such data-driven ROI was shown to be more powerful than a priori hypothesis or independent information and shall avoid increasing the Type I error rate^65^. Therefore, we averaged all individual interface and agent error trials from all subjects from both scenarios. On the AGAT of the Cz electrode we determined the times when the components had their maximal values for the positive components, and minimal for the negative. The time windows for searching the peaks were defined after visual inspection as follows: 100–200 ms for P200, 200–300 ms for N200, 250–350 ms for P3a, 400–450 ms for P3b, 400–500 ms for N450, and 500–650 ms for P600. The found peak times were used to display the topographical maps of the grand averaged data.

#### ErrP network dynamics

We projected the underlying sources from the data using Brainstorm^66^. The template MRI subject ICBM152^67^ was used for the forward model computation using symmetric boundary element method^68^. The head model was computed using cortex surface with adaptive integration and regular grid. The noise covariance matrix was computed for each subject individually on the preprocessed data. The sources were computed using unconstrained standardized low resolution brain electromagnetic tomography (sLORETA) (which according to the author’s findings has the lowest localization bias in the presence of measurement and biological noise^69^, and has one major limitation that only distinct fields with similar strength can be separated if simultaneously active^70^) and unconstrained dipole orientations. The inverse operator was applied on each grand averaged error trials for each subject individually. We used the Destrieux atlas^71^ to extract the activity of the anterior, mid-anterior and mid-posterior cingulate gyrus and sulcus from both left and right lobes.

#### Electrode selection and single-trial decoding of events and sources

The data between 180 and 570 ms capturing all components’ peaks was sub-sampled with an overlapping moving averaging filter of size 10, yielding 20 features per electrode. After standardizing, the data was reduced in dimensionality using Principal Component Analysis (PCA) by selecting the principal components that explained 95% of the original data variance. Several EEG-based BCI studies showed that SVM has good generalization properties, is robust in terms of low number of training data in comparison to high number of features, and outperforms other classifiers^72^. To analyze which signals SVM is isolating, we performed EEG-adapted forward wrapper feature selection for each subject individually. We balanced the number of trials for the classification by uniformly and randomly sub-sampling the class with more trials to equate the number of trials in the minority class. The classification in each iteration was done with 2-times-5-fold cross-validation. The data was randomly split in five subsets; for each validation, one subset was test data and the classifier was trained on the rest. This was repeated two times, each time the class with more trials was sub-sampled over-again.

**Figure 5:**
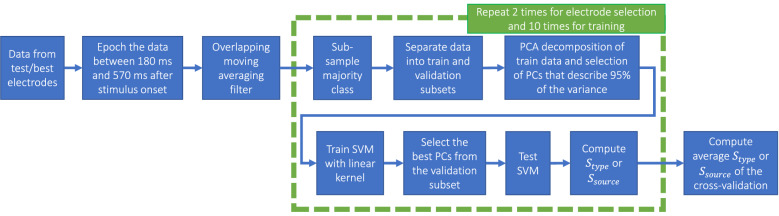
The pipeline for the electrode selection and the training of the SVM classifiers.

After the best electrodes selection, five linear-kernel SVM classifiers were trained for each subject individually: (1) error vs non-error events (from originally 26 electrodes × 20 features = 520 features, 95% of the variance was explained by $121.08\pm 15.22$ features), (2) agent vs interface errors (from originally 17 electrodes x 20 features = 340 features, 95% of the variance was explained by $82.438\pm 7.98$ features), (3) error vs non-error trials where the SVM classifier was trained only on the error trials made by the agent, and tested separately on agent error vs all non-error trials, and interface error vs all non-error trials; the data came from the electrodes that achieved the best $S_{source}$, (4) error vs non-error trials where the SVM classifier was trained only on the error trials made by the interface, and tested separately on agent error vs all non-error trials, and interface error vs all non-error trials; the data came from the electrodes that achieved the best $S_{source}$, and (5) three classes, one for non-error trials, one for agent errors, and one for interface errors; the data came from the electrodes that achieved the best $S_{source}$. The pipeline, shown in Fig. 5, for all classifiers was the same as previously described for the electrode selection, with the only difference that the testing of the classifiers was done with 10-times-5-fold cross-validation.

## Supplementary Information

Supplementary Information.

Supplementary Video 1.

Supplementary Legends.

Supplementary Video 2.

Supplementary Legends.

## Data Availability

The data is publicly available on https://github.com/VDimovaEdeleva/dataset-ErrP-Human-Agent-Collaboration.
