# Supplementary material for: Brain computer interface to distinguish between self and other related errors in human agent collaboration
Source: Sci Rep. 2022 Dec 1;12:20764. doi: 10.1038/s41598-022-24899-8 (PMC9715724; doi:10.1038/s41598-022-24899-8)
Supplement: Supplementary file 1 — Supplementary Information. [file 41598_2022_24899_MOESM1_ESM.pdf]

Brain Computer Interface to distinguish between self and other related errors in Human Agent Collaboration

Viktorija Dimova-Edeleva[1,*], Stefan K. Ehrlich[2], and Gordon Cheng[2]

[1]Technical University of Munich, Germany; Munich Institute of Robotics and Machine Intelligence (MIRMI)
[2]Technical University of Munich, Germany; TUM School of Computation, Information and Technology, Department of Computer Engineering, Institute of Cognitive Systems
[*]viktorija.dimova-edeleva@tum.de

Appendix

subject	electrode	impedance
**id1**	F8	87
	T8	311
**id2**	FC1	64
	F1	66
**id3**	C3	87
	P4	75
	O1	72
	F1	75
	C6	87
	C2	68
	F2	72
**id4**	O2	64
**id8**	C4	64
	Afz	61

Table A1. Impedances per subject that were above 60 kΩ

	raw data		after trial rejection	
	error trials	non-error trials	error trials	non-error trials
**id1**	510	1803	510	1803
**id2**	492	1710	492	1709
**id3**	546	1838	546	1838
**id5**	571	1830	544	1753
**id6**	529	1779	522	1757
**id7**	509	1763	507	1733
**id8**	488	1725	488	1717
**id9**	573	1867	556	1813
**id10**	489	1782	488	1771
**id11**	557	1864	552	1846
**average**	526.4	1796.1	520.5	1774
**std**	33.53	54.41	27.23	48.92

Table A2. Trials per subject before and after trial rejection

	raw data		after trial rejection	
	error trials	non-error trials	error trials	non-error trials
**id4**	547	1792	236	805

Table A3. Number of trials before and after trial rejection for subject id 4

Results from subject id4

Classification of single trials from subject id4

Using linear Support Vector Machine (SVM) classifier, 70.47%±2.5% of the error events and 71.27%±2.42% of the non-error events were correctly predicted for a chance level of 58.51% for an average of 94.4±0.55 observations per fold.

For the source of the error, 64.2%±4.6% of the agent errors and 65.3%±3.94% of the interface errors were correctly predicted, which is slightly above the chance level of 62.57% for 40 observations per fold.

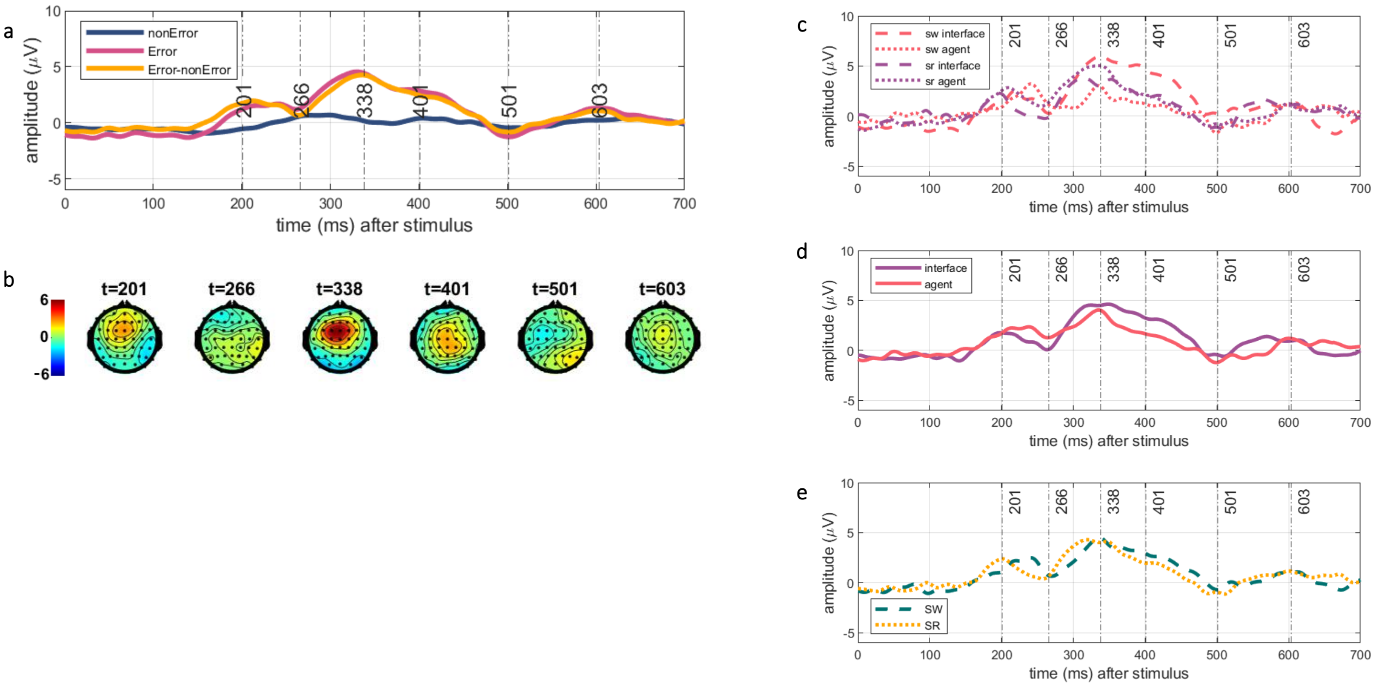

Figure A1. Averages from subject id4 on the Cz electrode: **a** the first positive peak of the difference between the averaged error and non-error trials can be observed around 201 ms, the first negative around 266 ms followed by two positive peaks around 338 ms and 401 ms, the next negative peak around 501 ms, and the last positive peak around 603 ms. **b** the topographic maps of the difference between the grand average of the error trials minus the grand average of the non-error trials for the positive and negative peaks on the Cz electrode. **c** the differences in the amplitude of the responses for the different sources of the errors in the different scenarios. **d** the response for the interface errors at the peaks has higher amplitudes compared to the response for the agent errors. **e** the observable differences in the amplitude for the responses in the different scenarios which are smaller in comparison to the differences in the responses for the different sources of errors.
