# Supplementary material for: Brain computer interface to distinguish between self and other related errors in human agent collaboration
Source: Sci Rep. 2022 Dec 1;12:20764. doi: 10.1038/s41598-022-24899-8 (PMC9715724; doi:10.1038/s41598-022-24899-8)
Supplement: Supplementary file 3 — Supplementary Legends. [file 41598_2022_24899_MOESM3_ESM.docx]

**Video S1.** An example video of a test block for the Shared Responsibility scenario. The subject can control the movement of the object when the next correct tile is upwards or to the right. The agent controls the movements when the next correct tile is downwards or to the left. Error events can be seen both when the subject and the agent control the movement of the object.
