# Supplementary material for: Brain computer interface to distinguish between self and other related errors in human agent collaboration
Source: Sci Rep. 2022 Dec 1;12:20764. doi: 10.1038/s41598-022-24899-8 (PMC9715724; doi:10.1038/s41598-022-24899-8)
Supplement: Supplementary file 5 — Supplementary Legends. [file 41598_2022_24899_MOESM5_ESM.docx]

**Video S2.** An example video of a test block for the Shared Workspace scenario. The subject is controlling the movement of the object on the blue tiles of the grid. Error events can be seen both when the subject and the agent control the movement of the object.
